# Tuberous sclerosis: a review of the past, present, and future

**DOI:** 10.3906/sag-2002-133

**Published:** 2020-11-03

**Authors:** Sanem Pınar UYSAL, Mustafa ŞAHİN

**Affiliations:** 1 Department of Neurology, Harvard Medical School, Boston Children’s Hospital, Boston Massachusetts USA

**Keywords:** Tuberous sclerosis, mTOR, autism, epilepsy, rapamycin

## Abstract

Tuberous sclerosis complex (TSC) is an autosomal dominant, multisystem disorder that is characterized by cellular and tissue dysplasia in several organs. With the advent of genetic and molecular techniques, mutations in the *TSC1* or *TSC2* genes were discovered to be responsible for mTOR overactivation, which is the underlying mechanism of pathogenesis. TSC is a highly heterogenous clinical entity with variable presentations and severity of disease. The brain, heart, skin, eyes, kidneys, and lungs are commonly involved in this syndrome, with neurologic symptoms comprising a significant source of morbidity and mortality. In 2012, the diagnostic criteria for TSC were revised by the International Tuberous Sclerosis Complex Consensus panel, and genetic testing was incorporated into the guidelines. Early detection of cardiac rhabdomyomas or TSC-associated skin lesions can suggest the diagnosis and underlie the importance of clinical vigilance. Animal studies have demonstrated the benefit of using mTOR inhibitors for various symptoms of TSC, and they have been successfully translated into clinical trials with significant improvement in symptom burden. Subependymal giant cell astrocytomas, renal angiomyolipomas, and epilepsy are the three FDA-approved indications in relation to TSC for the use of everolimus, which is a first generation mTOR inhibitor. Rapamycin has been FDA approved for lymphangioleiomyomatosis. Other TSC symptoms that could potentially benefit from this class of medication are currently under investigation. TSC constitutes a unique combination of protean physical symptoms and neurobehavioral abnormalities. TSC associated neuropsychiatric disorders (TAND), including intellectual disability, mood disorders, and autism spectrum disorder, represent significant challenges but remain underdiagnosed and undertreated. The TAND checklist is a useful tool for routine use in the clinical evaluation of TSC patients. A multidisciplinary treatment plan, based on the specific problems and needs of individuals, is the key to management of this genetic condition. Ongoing research studies have been providing promising leads for developing novel mechanistic strategies to address the pathophysiology of TSC.

## 1. Introduction

Tuberous sclerosis complex (TSC) is a rare, multisystem, autosomal dominant syndrome characterized by tumorigenesis and is associated with neurologic and behavioral abnormalities. The pathogenesis is driven by hyperactivation in the mTOR pathway due to de novo or inherited mutations in the *TSC1* or *TSC2* genes. TSC was first identified by German pathologist Friedrich Daniel von Recklinghausen, in 1862, in a baby with cardiac myotomas and sclerotic brain lesions who died shortly after birth [1].

It was better defined in 1880 by French neurologist Désiré-Magloire Bourneville as “tuberous sclerosis of the cerebral convolutions”; hence, the disease was named “Bourneville’s disease” after him. His patient reportedly had seizures, intellectual disability, and renal angiomyolipomas (AMLs) in the form of “hard masses, one the size of a walnut” [2]. In 1908, another German neurologist, Heinrich Vogt, established the Vogt triad for TSC, comprising intellectual disability, intractable seizures, and facial angiofibromas [3]. The first use of the term tuberous sclerosis complex was by Sylvan Moolten in 1942 [4]. In 1972, Spanish-American neurologist Manuel Rodriguez Gomez established the first diagnostic criteria for TSC, and he has since been viewed as the father of TSC in the USA [5]. 

**Figure 1:**
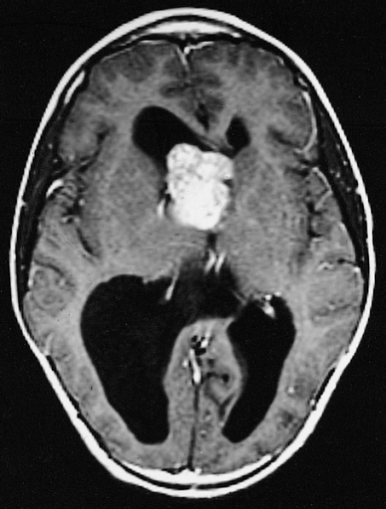
MRI of a SEGA (Image courtesy of Wikimedia Commons [online]. Website https://commons.wikimedia.org/wiki/File:MRI_of_brain_with_subependymal_giant_cell_astrocytoma.jpg [accessed 05 January 2020].).

**Figure 2:**
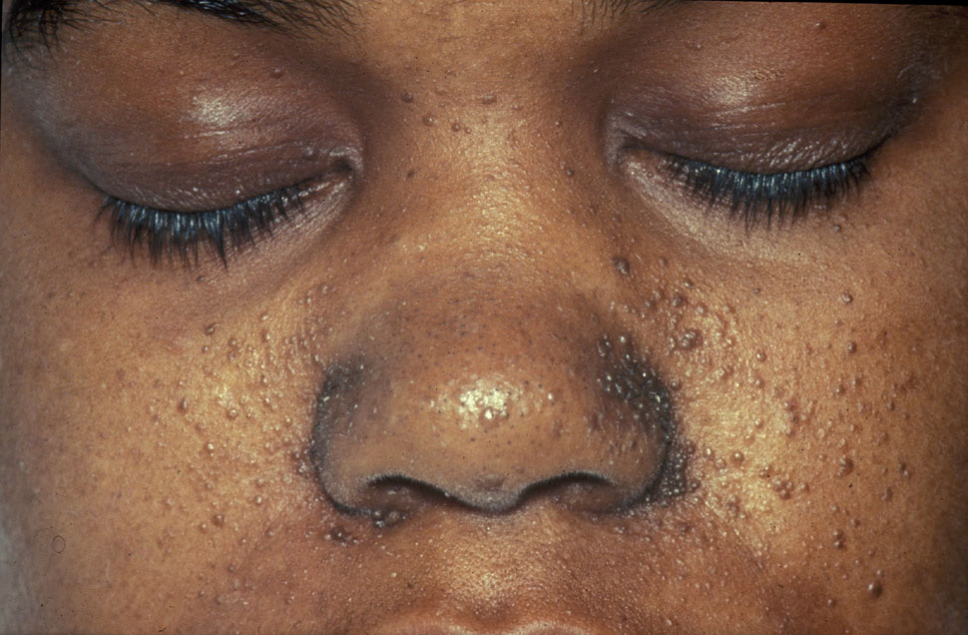
Facial angiofibromas (Image courtesy of Wikimedia Commons [online]. Website https://commons.wikimedia.org/wiki/File:Patient_with_facial_angiofibromas_caused_by_tuberous_sclerosis.jpg [accessed 05 January 2020].).

Even though there are no pathognomonic signs for TSC, various clinical stigmata are commonly seen as part of the syndrome, which raise suspicion for the diagnosis. Common manifestations include cortical tubers, subependymal nodules, subependymal giant cell astrocytomas (Figure 1), seizures, cardiac rhabdomyomas, renal AMLs, retinal hamartomas, pulmonary lymphangioleiomyomatosis (LAM), facial angiofibromas (Figure 2), ash-leaf spots, shagreen patches, intellectual disability, and autism spectrum disorder [1,3]. Once the diagnosis is suspected, genetic testing can be performed to look for mutations in the *TSC1* or *TSC2* genes and guide genetic counseling. As a matter of fact, improvements in the realm of genetics opened a new era for TSC in the 1990s, when the *TSC1* and *TSC2* genes were identified, which led to the exploration of the molecular pathways involved [6].

*TSC1* and *TSC2* genes encode the proteins hamartin and tuberin, respectively. These proteins compose the TSC complex, which acts as a brake on the mTOR signaling cascade [7,8]. In agreement with this, mTOR inhibitors, such as rapamycin and its analogs (rapalogs), are commonly used in a clinical setting for various symptoms of this condition [9]. The current management of TSC is mostly symptomatic, with pharmacologic, surgical, or behavioral intervention options. Due to the vast phenotypic heterogeneity encountered, not all therapeutic approaches benefit the entirety of symptoms or patients to the same extent; hence, a personalized treatment strategy is critical [10]. 

## 2. Epidemiology

The incidence of TSC has been estimated as occurring in 1/6000–10,000 newborns annually, and therefore, it is categorized as a rare disease. It affects approximately 2 million people globally [11]. The prevalence in Europe is approximately 11,500–25,000 [12]. There is no sex or ethnicity predilection [13]. However, differences in sex predominance have been observed in numerous symptoms (see the Clinical presentation section). In 2012, a conference was held in Washington, DC, USA, by the International Tuberous Sclerosis Complex Consensus group to revise the diagnostic criteria for TSC, providing a comprehensive list consisting of major and minor criteria for use by physicians in a clinical setting [14,15] (Table). 

**Table:** TSC diagnostic criteria.

**Diagnostic criteria according to the 2012 International Tuberous Sclerosis Complex Consensus Conference** **Definite diagnosis:** Two major features, or 1 major feature with greater than 2 minor features, or the presence of a TSC1 or TSC2 mutation of confirmed pathogenicity **Possible diagnosis:** Either 1 major feature or greater than 2 minor features **Major criteria:** Skin/oral cavity • Hypomelanotic macules (n > 3, at least 5 mm in diameter) • Angiofibromas (n > 3) or fibrous cephalic plaque • Ungual fibromas (n > 2) • Shagreen patch Central nervous system • Cortical dysplasias (includes tubers and cerebral white matter radial migration lines) • Subependymal nodules • Subependymal giant cell astrocytoma Heart • Cardiac rhabdomyoma Lungs • Lymphangioleiomyomatosis Kidney • Angiomyolipoma (n > 2) Eyes • Multiple retinal hamartomas **Minor criteria:** Skin/oral cavity • Confetti skin lesions • Dental enamel pits (n > 3) • Intraoral fibromas (n > 2) Kidney • Multiple renal cysts Eyes • Retinal achromic patch Other organs • Nonrenal hamartomas **Genetics:** Identification of either a TSC1 or a TSC2 pathogenic mutation in DNA from normal tissue is sufficient to make a definite diagnosis

## 3. Molecular genetics

TSC is caused by mutations in either of the *TSC1* or *TSC2* genes, which were discovered through the use of *Drosophila* models in the 1990s [16]. *TSC1* is found on chromosome 9 (9q34) and *TSC2* is found on chromosome 16 (16p13.3), encoding the proteins hamartin and tuberin, respectively [7,8]. TSC is also genetically linked to autosomal dominant polycystic kidney disease, as the *PKD1* and *TSC2* genes are closely located (48 base pairs apart) on chromosome 16. When both genes are affected due to a contiguous gene deletion, it may lead to a clinical picture called PKD-TSC (MIM #600273) with severe renal symptoms [17].

TSC can arise due to *de novo* or inherited mutations. *De novo* mutations constitute two-thirds of all TSC diagnoses. The remaining one-third is inherited in an autosomal dominant fashion [18]. The genetic pattern of TSC can be described by Knudson’s 2-hit hypothesis, where the acquisition of a somatic mutation in a previously functional allele of *TSC1* or *TSC2* , in addition to the existing germline mutation in the other allele, leads to the disease state [19]. Most *TSC2* cases are sporadic and have more severe manifestations, while the ratio of *TSC1* to *TSC2* mutations in familial cases is close to one [20]. TSC shows almost complete penetrance with wide phenotypic variability. This means that any individual carrying a TSC mutation would be afflicted with the disease, but to varying degrees. The large number of possible mutations in the TSC genes also contributes to the heterogeneity within the patient population [21]. 

The *TSC1* and *TSC2* genes differ from each other in that *TSC1* mutations are mostly nonsense or frameshift mutations, leading to protein truncation, whereas missense mutations, large deletions, or rearrangements are seen more with *TSC2* [22]. Additionally, *TSC1* mutations have been identified in ~10–20% of patients clinically diagnosed with TSC, while *TSC2* mutations have been identified in ~70–90% [23,24]. Several thousand small mutations have been shown to cause TSC, which can be found in the online Leiden Open Variation Database http://chromium.lovd.nl/LOVD2/TSC/home.php. However, genetic testing may be negative in 10–25% of inherited cases due to reasons such as somatic mosaicism, or mutations in intron or promoter regions [25]. 

The protein products of the TSC genes, hamartin and tuber, work together within the same intracellular pathway, which explains why a mutation in either gene can give rise to the same disease. The downstream target of these proteins is the mammalian target of rapamycin complex 1 (mTORC1), which is a protein serine/threonine kinase complex involved in many important anabolic and catabolic processes, such as translation, cellular growth, proliferation, stress response, and autophagy [9,26,27]. 

## 4. Pathophysiology

**Figure 3:**
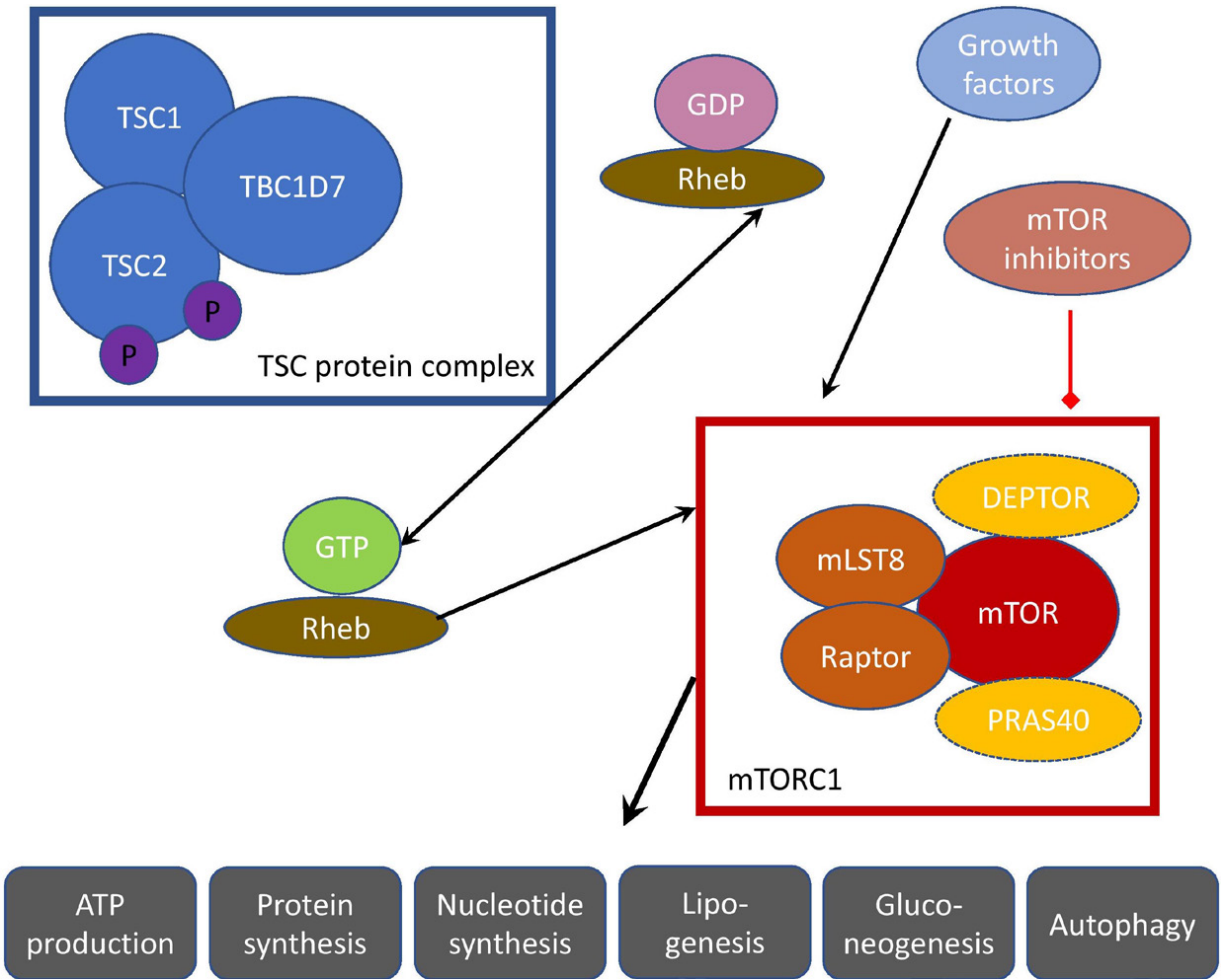
mTOR signaling pathway.

mTORC1 is a protein complex that contains mTOR, a rapamycin-associated protein of TOR (raptor), and mLST8 [28] (Figure 3). The major driver of the cellular hyperplasia and tissue dysplasia seen in TSC is the overactivation of the mTORC1 signaling pathway. Hamartin and tuberin bind to a third protein, TBC1D7, to form the TSC protein complex as part of this cascade [29]. The heterotrimeric TSC complex acts as a GTPase-activating protein for RAS homologue enriched in brain (Rheb), which is the functional mediator between the TSC complex and mTORC1 [30]. Under normal circumstances, the TSC complex functions to keep it in an inactive GDP-bound state, thus rendering Rheb unable to stimulate mTORC1.

When second hit mutations affect either *TSC1* or *TSC2* , the brake on Rheb by the TSC complex is released as the complex can no longer be formed [9]. Therefore, mTORC1 is constitutively activated by Rheb, regardless of the upstream signals. The mechanism by which Rheb regulates mTORC1 still needs exploration. Rheb-induced mTOR activation results in the stimulation of S6 kinase and inhibition of 4EBP1, the eukaryotic translation initiation factor 4E-binding protein 1, leading to unrestricted protein synthesis and proliferation [31].

The mTOR pathway can be pharmacologically manipulated by rapamycin (Sirolimus), which binds to FKPB12 and causes the mTORC1 complex component raptor to dissociate, rendering mTORC1 unable to stimulate the downstream targets of anabolism [32,33]. Rapamycin was discovered in the 1970s on Easter Island and was originally used as an antifungal compound [34]. Later, it drew further attention due to its immunosuppressant and antiproliferative properties, causing it to become an important agent in oncology. In the early 2000s, rapamycin was demonstrated to be effective for symptom control in TSC mouse models by several labs, and the bench-to-bedside studies that followed [35,36]. 

The activity of mTORC1 is also influenced by the upstream components of several intersecting pathways. mTORC1 is negatively regulated by PRAS40, the proline-rich Akt substrate of 40 kDa and DEPTOR, the DEP domain containing mTOR-interacting protein, which works as a component of the GATOR complex. [37]. In addition to growth-inducing states in the presence of sufficient energy and nutrients, mTORC1 is also turned on by tyrosine kinase growth factor receptors, such as insulin, insulin-like growth factor 1, brain-derived neurotrophic factor, and epidermal growth factor receptor [38]. This results in the activation of the Ras/ERK and PI3K/Akt signaling pathways, which converge on the TSC complex and cause its activation favoring a progrowth state [39]. 

mTORC1 has been demonstrated to regulate both anabolic and catabolic processes. When activated, it stimulates protein synthesis, nucleotide synthesis, gluconeogenesis, lipogenesis, and ATP production. When it is turned off, it inhibits cell growth through several mechanisms including autophagy [40,41]. mTORC2 complex is less well understood, especially in terms of its role in the pathogenesis of TSC [42]. It is involved in the regulation of cytoskeletal dynamics. Additionally, it is not sensitive to the effects of acute rapamycin treatment because the core component, RICTOR, does not bind FKBP12 [43]. Further studies could help to characterize its functions and explore the possibility of interactions with the mTORC1 pathway. 

Both mTOR and Rheb are ubiquitous in the body, causing multisystem involvement in the presence of TSC mutations [43]. Thanks to the successful implementation of genetic and molecular discoveries into clinical settings, TSC serves as model for many other diseases involving similar pathways or cellular processes [44].

## 5. Clinical presentation

The clinical manifestations of TSC are protean in terms of severity and the range of tissues it can involve. Despite the potential of TSC to involve any organ system in the body, some organs are more affected than others. Neurologic manifestations, including seizures and cognitive impairment, are the primary source of patient and caretaker burden, followed by renal abnormalities [45,46]. 

Although there is no single symptom specific to TSC, a constellation of findings on physical examination and imaging raises the suspicion for diagnosis [47]. The revision of the diagnostic criteria for TSC by the International Tuberous Sclerosis Complex Consensus group incorporated genetic testing into the clinical framework of TSC [14]. Even before genetic testing is undertaken, the presence of a first-degree relative with TSC puts the patient at 50% risk for having the disease [48]. 

The 2012 diagnostic criteria list includes major and minor features, determining a TSC diagnosis to be categorized as definite or possible (Table). A definite diagnosis can be made when 2 major or 1 major and 2 minor criteria are fulfilled. Patients with 1 major or 2 and more minor criteria meet the criteria for having a possible TSC diagnosis. A thorough clinical evaluation should be followed by genetic testing for confirmation of the disease and prognostication [45].

### 5.1. Neurologic manifestations

The neurologic issues of TSC comprise the most important cause of impairment in the majority of patients, owing to their prevalence and the severity of symptoms [46]. The array of manifestations include epilepsy, cortical tubers, subependymal nodules and giant cell astrocytomas, intellectual disability, autism spectrum disorder, and behavioral problems [49,50]. The foremost neurologic symptom in TSC is epilepsy, afflicting 90% of patients [51].

The onset of epilepsy is variable, but most patients present before the age of 1 year [52]. All seizure types can be seen with TSC. The most common seizure type in early life is infantile spasms, which affects nearly 40% of patients with TSC-associated epilepsy [53]. Although earlier studies supported the hypothesis of seizures originating from cortical tubers, the exact origin and mechanism of epileptogenesis are still debated [54]. Several studies have demonstrated a lack of correlation between tuber burden and epilepsy severity [55]. Up to 38–50% of seizure patients are refractory to the point of necessitating surgical intervention [56]. The age of onset and severity of seizures are most predictive of long-term cognitive and behavioral outcomes [57,58]. 

Cortical tubers (80–90%) are one type of brain malformation that present as part of TSC and give the disease its name. They are thought to be caused by a failure of cellular differentiation and neuronal migration during neurodevelopment [59]. Radial migration lines occur due to a similar process and can be observed with the tubers [45]. The cortical tubers contain giant dysplastic neurons and astrocytes, and they tend to stay stable in size [60]. Microtubers may also be found in normal appearing white matter [61]. 

Subependymal nodules (SENs) are formations that arise mostly along the lateral and third ventricle walls, and are seen in >80% of patients. Approximately 5–15% of these growths transform into subependymal giant cell astrocytomas (SEGAs) [47]. SEGAs are composed of ganglion-like giant cells expressing both neuronal and astrocytic markers [62]. SEN/SEGAs may remain asymptomatic [51]. However, if they are located at the foramen of Monro, they can potentially cause obstructive hydrocephalus and increased intracranial pressure. Both SENs and SEGAs can be detected prenatally or at birth, and it is rare for SEGAs to grow after the age of 20 [14]. Most of these lesions tend to progressively calcify [63]. 

### 5.2. Renal manifestations

Renal abnormalities can also lead to significant morbidity and mortality, as they are commonly encountered in TSC patients, and can lead to complications if left untreated [64]. The most common renal lesion is angiomyolipoma, a hamartoma composed of blood vessels, smooth muscle, and adipose tissue [65]. They are often bilateral and multiple. [66]. Although benign in nature, these lesions have a tendency to bleed, and therefore should be watched closely for timely intervention. In severe cases, renal AMLs may lead to renal failure [67]. 

The second most frequent lesion in the kidney is single or multiple simple cysts. They are seen in 45% of patients and can result in hypertension or kidney failure [68]. The combination of AMLs and cysts in the same patient is highly suspicious for a TSC diagnosis [69]. In early-onset severe cases, they may constitute stigmata of PKD-TSC syndrome (see also the Genetic background section). 

### 5.3. Dermatologic manifestations

The dermatologic lesions seen with TSC are of paramount value, as their presence heralds the diagnosis in a considerable number of cases [70]. Many of the major features listed in the 2012 diagnostic criteria for TSC included cutaneous manifestations. Among these, hypomelanotic macules or ash-leaf spots, which were named after the European Mountain Ash Tree, can be seen in up to 90% of patients [71]. They are detected at birth or during early infancy. They may be difficult to visualize especially in fair-skinned individuals or small babies; therefore, the use of ultraviolet light (Wood’s lamp) can be helpful [51]. 

Another important dermatologic finding is facial angiofibromas or adenoma sebaceum. These lesions are comprised of connective and adipose tissue and are found in 75% patients over 9 years of age [71,72]. They appear in the central face and increase in number with age, which is a common source of concern [73]. TSC patients can also present with fibrous plaques in the forehead (20%), shagreen patches in the lumbosacral region (20%), and ungual or gingival fibromas (20%) [74].

### 5.4. Cardiac manifestations

Intracardiac rhabdomyomas are seen in nearly 50% of patients [75]. They are one of the earliest TSC lesions to emerge as the cardiac rhabdomyomas can be detected on prenatal ultrasound as early as 22 weeks of gestation [11]. On average, the lesions tend to cluster as a few lesions, grow to a size of 3–25 mm, and are mostly found in the ventricles of the heart along the septum. They tend to regress within 3 years of life [76]. Although rare, some rhabdomyomas may lead to arrhythmia, valvular defects, or cardiac failure, so prenatal and postnatal surveillance are critical until regression [77].

### 5.5. Pulmonary manifestations

Pulmonary involvement is much less common than the previously mentioned manifestations of TSC. The most frequent pulmonary lesion is LAM, which is almost exclusively seen in adult females [78]. This suggests that the pathogenesis may be driven by estrogen, which has also been evidenced by animal models of LAM [79]. Infiltration of the lung tissue by smooth muscle cells is characteristic of LAM, leading to cystic lung changes and potential complications of pneumothorax, pleural effusion and hemoptysis [52]. Multifocal micronodular pneumocyte hyperplasia (MMPH) is another type of pulmonary lesion associated with TSC [80].

### 5.6. Ophthalmic manifestations

The eye is another organ that can be commonly affected in TSC, as they arise from ectoderm like the central nervous system. Retinal astrocytic hamartomas are seen in 35–50% of patients and are typically benign unless they compress the optic disc [81]. Additionally, areas of hypopigmentation around the retina called retinal achromic patch can be observed in 39% of TSC patients [82].

### 5.7. Other manifestations

TSC can involve the gastrointestinal system (liver AML, colon polyps, or hamartomas), thyroid, pituitary gland, pancreas, and gonads (AML, fibroadenoma) [83]. However, the number of cases reported in the literature is limited.

### 5.8. Tuberous sclerosis associated neuropsychiatric disorders (TAND)

The group of cognitive and behavioral problems associated with TSC causes a great burden, both for TSC patients and their caretakers. Most neuropsychiatric symptoms of TSC present a challenge for treatment and ironically do not receive as much attention as the physical stigmata of the disease [84]. The gap between burden and treatment exhibited for the tuberous sclerosis associated neuropsychiatric disorders (TAND) was found to be similar to those seen in the approach to HIV, where the major focus is also on the physical symptoms [85]. The umbrella term TAND was coined through the inspiration by HAND, which defines the HIV-associated neurocognitive disorders [86].

Among the neuropsychiatric manifestations of TSC, ASD has a special place, both in terms of clinical approach and research focus. Approximately 26–50% of TSC patients fulfill the criteria for autism spectrum disorder (ASD) [87]. ASD in TSC has many overlaps with idiopathic ASD, which is another factor that makes TSC an important genetic model to study this condition [88]. It has been observed that TSC patients with ASD tend to have a more severe epilepsy phenotype, in line with several studies demonstrating that poorly controlled seizures contribute to worse cognitive outcomes [89]. Overall in TSC, there is a wide range of severity for intellectual disability and patients are affected by the neurologic comorbidities at varying degrees [90]. The exact nature of the relationship between autism, intellectual disability, and epilepsy needs further investigation.

TAND is a broad category of symptoms encompassing multiple dimensions of cognitive, psychological, and social issues encountered within the context of TSC. The 2012 International Tuberous Sclerosis Complex Consensus group established the guidelines for evaluation of neuropsychiatric manifestations of TSC, by providing the practitioners with a TAND checklist. This proved to be a practical clinical tool for practitioners to address TAND, which are often missed and therefore undertreated. Properly addressing TAND can dramatically improve the quality of life of TSC patients and their families [91].

The multiple levels covered in the TAND checklist included the following: behavioral level (mood swings, self-injury, obsessions, aggression, impulsivity, eating, and sleeping difficulties), psychiatric level (autism spectrum disorder, attention deficit hyperactivity disorder, anxiety, and depression), intellectual level (IQ assessments and adaptive behaviors; e.g., daily living skills), academic level (reading, writing, spelling, and mathematics) and psychosocial level (quality of life, self-esteem, parental stress, and relationship difficulties) [84]. The different levels provide a common ground between patients and physicians to have a conversation and come up with a personalized management plan. This is of great importance, as up to 90% of TSC patients are observed to have TAND features during at least one period of their life [92].

One should bear in mind that the TAND checklist was not designed to be used as a rating scale, but rather a tool to make an individual action plan based on the TAND profile [84]. Neuropsychiatric reevaluations are recommended by the 2012 TSC guidelines, as the profile of the individual may change over time. Sudden changes in behavior should prompt evaluation for underlying medical causes, such as new brain lesions or seizures [93].

## 6. Treatment options

As a consequence of the multisystem involvement in TSC, each symptom demands evaluation and management within the relevant clinical context. mTOR inhibitors have been groundbreaking in the TSC world due to their ability to target the molecular defect in the disorder. However, animal models and clinical studies have shown that not all TSC-related symptoms benefit from mTOR inhibitors to the same extent [94]. Research is still ongoing for the optimization of mTOR inhibition for each symptom and what other pharmacologic and nonpharmacologic approaches could be employed for tackling the challenges in TSC. In particular, the timing of treatment may be crucial for the neurocognitive symptoms, and treatment during early critical windows may be necessary [95]. Regardless of the choice of intervention, genetic counseling should be included in the discussion with families.

### 6.1. Neurologic management

Once a diagnosis of TSC is reached, a baseline MRI is recommended to look for the presence of any cortical malformations as tubers, SENs, or SEGAs [68]. Surgery and mTOR inhibitors are the current treatment options for asymptomatic SEGAs; however, surgical intervention is recommended in acute cases [91]. After the success of the EXIST-1 clinical trial, the FDA approved the use of everolimus for individuals with tuberous sclerosis who present with SEGAs that are not amenable to surgery [96].

As mentioned previously, epilepsy remains one of the primary sources of morbidity for TSC patients. Early intervention is key for both better control of seizures and improved neurocognitive outcomes. For infantile spasms, vigabatrin is the best choice of medication, and ACTH is an alternative in cases with insufficient response [97]. Vigabatrin is an irreversible inhibitor of GABA transaminase. It helps to increase the GABA concentration and potentially reset the imbalance between GABA and glutamate neurotransmitters, which is a proposed mechanism of epileptogenesis [98]. Patients should be counseled about the possible side effects of retinal toxicity and evaluated for vision changes. 

Most antiseizure medications can be used for epilepsy in TSC. Alternative or complementary therapeutic options include surgery, ketogenic low glycemic index diet, and vagal nerve stimulation. Despite the presence of these modalities, refractory epilepsy is still a big concern, with seizures persisting in more than 60% of patients [53]. The EXIST-3 trial demonstrated the benefit of everolimus in patients with treatment-resistant focal seizures [99]. 

Neuropsychiatric conditions associated with TSC should be managed with a multidisciplinary team, focusing on the individual’s level of psychosocial and neurocognitive functioning. Despite contributing greatly to the burden of care, TAND has received little clinical attention, with less than 20% of cognitive and behavioral symptoms being treated [84]. During clinical visits, the TAND checklist is a useful diagnostic tool to determine which neuropsychiatric symptoms require special attention. An individualized educational plan should be established for school-aged patients [14].

Animal studies have shown correlations between seizure frequency and the extent of social deficits, which are improved by the early treatment of epilepsy [100]. Several clinical trials have aimed to investigate the relationship between mTOR inhibition and neurocognition. In a 6-month clinical trial of an mTOR inhibitor (everolimus) in children and adolescents with TSC (6–21 years of age), no improvement was detected in the active drug group when compared to those taking a placebo [101]. Another trial in Europe with a similar age group also failed to show an effect on cognitive abilities or neuropsychological functioning [102]. 

While there are several possible explanations for these results, one important aspect may be the age of treatment. It should be kept in mind that infancy is a critical course of the disease, where pharmacologic interventions may also lead to long-lasting unfavorable changes [103]. Therefore, determining the optimum therapeutic window is crucial [88]. A randomized clinical trial of early intervention with vigabatrin to prevent seizure development in TSC (EPISTOP) recently concluded in Europe, and an National Institutes of Health-funded trial to prevent epilepsy and improve neurocognitive outcomes in infants with TSC (PREVeNT) is currently ongoing in the USA.

### 6.2. Nonneurologic management

The EXIST-2 trial demonstrated the benefit of mTOR inhibitors for renal AMLs, and everolimus was approved by the FDA for asymptomatic and growing renal AMLs larger than 3 cm [104]. Second-line treatment options in cases with unsatisfactory response or a lack of access to everolimus include selective arterial embolization or radiofrequency ablation, especially for hemorrhaging or compressive lesions [105]. Sirolimus was approved by the FDA for use in patients with LAM in 2015, after obtaining successful results in the MILES trial [106]. Pulmonary function and capacity need to be regularly monitored for signs of clinical improvement. Last, but not least, topical rapamycin may be used for facial angiofibromas in cases of significant disfigurement or psychological stress [107].

For most TSC-related hamartomas, lifelong treatment will continue to be necessary, as many lesions tend to regrow and seizures may recur upon the discontinuation of medication [15,108]. Reported side effects of mTOR inhibitors include stomatitis, menstrual irregularities, acne, hyperlipidemia, infections, and poor wound healing, which are related to suppression of the immune system and changes in cellular metabolism [109–111]. 

In TSC, an interdisciplinary approach with consultations from neurologists, cardiologists, nephrologists, pulmonologists, psychiatrists, psychologists, social workers, educational specialists, genetic counselors and additional practitioners, based on the needs of the specific individual, is essential. To achieve this goal, TSC clinics have been established in numerous hospitals across the USA. TSC has been one of the great examples in medicine where the bench research has been successfully translated to improve the diagnostic and therapeutic yield in a clinical setting.

More recent studies have started to focus on developing alternative strategies for the treatment of TSC. One notable approach is the elimination of TSC-deficient cells through the induction of autophagy or oxidative stress by exploiting the inadaptability of the overactivated mTOR pathway [112–115]. Such research questions will continue to be explored and can be promising for the development of preemptive therapy for TSC. 

## 7. Conclusion

TSC is a rare genetic disorder characterized by hamartoma formation in multiple organs. The pathogenesis is driven by uncontrolled mTOR activation, which is the target of rapamycin and rapalogs to control the disease symptomatology with varying success. TSC serves as a model for epilepsy, autism, and tumorigenesis and many other diseases involving the mTOR pathway. 

Despite the morbidity and mortality that TSC symptoms are associated with, the medical world is fortunate for the discoveries into the genetic and molecular aspects of this disease. Owing to the immense endeavors of TSC researchers, new therapeutic options targeting vulnerabilities in the TSC-related pathways will continue to be developed that maximize efficacy and minimize toxicity. Biomarkers to demonstrate disease progression and treatment efficacy need to be identified for maximizing the benefit for all patient profiles. An individually tailored multidisciplinary approach, with special attention to cognitive and psychosocial comorbidities, is the key to success in the management of this disease. 

## Disclosures

Mustafa Sahin reports grant support from Novartis, Roche, Pfizer, Ipsen, LAM Therapeutics, and Quadrant Biosciences. He has served on Scientific Advisory Boards for Sage, Roche, Celgene, Aeovian, and Takeda.
